# Social space and alcohol use initiation among youth in northern Tanzania

**DOI:** 10.1371/journal.pone.0202200

**Published:** 2018-09-07

**Authors:** Haika Osaki, Gerry Mshana, Doris Mbata, Saidi Kapiga, John Changalucha

**Affiliations:** 1 National Institute for Medical Research, Mwanza, Tanzania; 2 Mwanza Intervention Trials Unit, Mwanza, Tanzania; AUSTRALIA

## Abstract

**Introduction:**

Alcohol use is a key risk factor for disease worldwide. Consumption of alcohol is increasing in sub Saharan Africa, where youth are already at high risk of HIV due to its high prevalence in the region. Studies show that youth begin drinking alcohol early; however, there is a need to further explore the initiation of alcohol use in order to design appropriate interventions in this population.

**Methods:**

We conducted a qualitative study with youth in Mwanza and Kilimanjaro regions in Tanzania to explore alcohol consumption among youth. Participants were a purposive sample of youth aged 15–24 composed of secondary school and college students, and formal and informal sector employees. We conducted 35 in-depth interviews using a semi-structured guide to understand youth’s personal experiences with alcohol consumption. Two social scientists conducted a multi stage, inductive analysis of the data.

**Findings:**

Alcohol consumption was reported to mainly start during adolescence, although in some cases it started as early as at 10 years of age. Young women reported drinking less, and initiated drinking later compared to males. Social space assumed a primary role in alcohol initiation. The social environment and influence of important social actors were key aspects of youth’s social space. Youth reported starting to consume alcohol at home, social events and in stressful environments with key influencers being parents, relatives, peers and intimate partners.

**Conclusions:**

Our findings show that the social space (social environment and interactions) plays an important role in influencing youth initial consumption of alcohol. Interventions addressing alcohol initiation among the population need to address the social spaces where initiation takes place and engage the significant actors in these spaces. There is need to further explore underlying societal drinking norms to better understand how they shape social environments and young people’s initiation of alcohol use.

## Introduction

Alcohol use is a major social and public health problem and an important risk factor for disease worldwide. Alcohol use is a risk for communicable [1–4] and non-communicable diseases including cancers, cirrhosis and mental illness [5]. Compared to other regions, youth in sub Saharan Africa (SSA) are at an increased risk of contracting HIV [6]. Alcohol use furthers this risk as it is linked to risky behavior such as casual and unprotected sex, transactional sex and multiple and concurrent sexual partnerships [1–4, 7, 8].

In Africa, alcohol consumption is increasing in the population [9, 10]. For most of the 20^th^ century, per capita alcohol consumption in Africa was among the lowest of any of the WHO world regions. Although most Africans still practice abstention, the region has the highest prevalence of heavy episodic drinking in the WHO region with 46% of women and 59% of male drinkers engaging in weekly heavy episodic drinking [11]. Alcohol use and Alcohol Use Disorders (AUDs) consequently account for 2.4% of deaths each year [12]. A systematic review of alcohol use among youth (aged 15–24 years) in East Africa showed that, 47–70% of males and 24–54% of females reported ever using alcohol while 20–45% of males and 12–47% of female reported current alcohol use [10].

Studies on alcohol consumption in developed countries show that youth begin drinking early, often during adolescence [13–15]. Early onset of alcohol use is likely to prolong lifetime consumption and subject the user to an increased progression of AUDs [16]. In Tanzania, youth live in an environment where alcohol is widely sold and promoted [17] with little implementation of the minimum legal age for drinking [18]. Like in other SSA countries, the initiation of alcohol use and its determinant factors have not been widely documented in Tanzania [10]. There is a need to explore factors influencing alcohol use initiation among youth to better understand their perceptions, experiences and motivations for using alcohol and inform interventions that aim to prevent or delay alcohol use in this population.

Society and social space: a social ecology of youth alcohol initiation

In this article, we use a social ecological model to interpret the complex relationship between social and physical space in the context of youth alcohol initiation in Northern Tanzania. The main premise of this model is that human development takes place within a set of layered and dynamic environments and that, the existing complex relations within and between these environments shape behavior [19]. We use Urie Bronfenbrenner’s (1979) levels of environmental influence to explain factors influencing youth alcohol initiation among the study population [20]. Bronfenbrenner categorizes environmental influences on behavior into three main categories: microsystems which consist of interactions among family members, peers and other interpersonal influences; mesosystems which consist of physical, family and school settings and exosystems which consist of the larger social system of economics [20].

Studies exploring the initiation of alcohol use and circumstances of young people’s first alcohol use mainly point to interpersonal influences such as peers, parents and other adults [13, 14, 21]. These ‘actors’ are part of the environment where young people’s social development takes place. This social environment is composed of physical and social space. Members of society interact in various ‘social spaces’ some of which play a significant role in shaping or constraining young people’s behavior towards alcohol use. Social space is defined as the invisible set of social and cultural relationships that translate to and are shaped by physical space [22]. Physical space is the setting where these relationships take place [23, 24].

Social space, although related to physical space, is different in that it connotes a relational rather than a geographical location [22]. According to Bourdieu, social space is a metaphor for our experiences and relationships in social life [24]. Physical space points to the site where social actors are positioned and where interaction takes place, in this case, the physical locations where young people learn and start to consume alcohol.

In this paper, we explore the influence of socialization and social space on the initiation of alcohol use among young people. We present findings from a qualitative study conducted in northern Tanzania. Our study explored young people’s perceptions about alcohol use and their drinking experiences. Alcohol use initiation among youth emerged as a strong theme in our findings. Subsequently, we recommend interventions to prevent or delay alcohol use among the population.

## Methods

### Sampling and recruitment

We conducted this study in Mwanza and Kilimanjaro regions in Tanzania. The two regions were purposively selected because of their contrasting drinking cultures where Kilimanjaro has more permissive norms towards youth alcohol use and higher alcohol consumption compared to Mwanza [17]. Both regions were favorable as they have a wide presence of industries, educational institutions and plantations for the recruitment of students and youth in the formal sector.

We purposively selected five districts; three from Mwanza and two from Kilimanjaro, to conduct the study. The districts provide urban rural contrasts in addition to the geographical variation. In Mwanza, Nyamagana district represented the urban area and Magu and Sengerema districts represented the rural areas whereas Moshi urban and Moshi rural districts were selected from Kilimanjaro.

This study consisted of a quantitative and qualitative component; this paper focuses on the qualitative component. Prior to the qualitative component, we conducted a survey for a cross sectional study with young people aged 15 to 24 who were secondary school students, college students, formal sector employees and casual laborers [17]. For secondary school students, we secured a list of all government and private secondary schools in the district. We then randomly selected two schools from each district and then selected two classes from each school (excluding final year students who were preparing for examinations). Thereafter, we randomly selected 16 boys and 16 girls from each class to participate in the survey. In colleges and universities; we randomly selected two colleges per district. For each institution, we obtained a list of courses and programs and randomly selected two for inclusion in the study. From each program, we then selected 16 men and 16 women to also take part in the survey.

Industrial employees were selected from all identified non-alcohol producing industries and had to be employed for at least six months. Industries included bottling factories, cotton ginneries and metal processing workshops. After contacting employers and informing them about the study, they shared a list of eligible employees. We surveyed all employees who consented to participate in the study. For casual laborers, survey participants included all casual laborers who were identified from construction sites, car workshops and plantations within all districts in Mwanza region and rural and urban Moshi districts in Kilimanjaro.

After completing the survey for the quantitative part, we used this sample to purposively select a sample of respondents to participate in Participatory Group Discussions (PGD). From the sample of participants who were selected to take part in PGDs, we selected those who provided rich and detailed information during discussions to take part in further In-depth Interviews (IDIs). In order to balance our sample and ensure a collection of a broad range of experiences, we selected participants who had reported both currently taking alcohol and not taking alcohol in questionnaire interviews conducted during the cross sectional study.

### Data collection

IDIs explored personal perceptions and experiences regarding alcohol consumption and its related risks. One same sex researcher conducted the interview in Swahili (national language) using a semi-structured guide. The interviewer and interviewee first discussed and agreed on a time for the interview. Interviews took place in private locations that were conducive to allow participants to speak freely and avoid any concerns about violating confidentiality. Interviews were audio recorded after obtaining consent from the participant.

### Data analysis

After data collection, all transcripts were coded using NVivo 10 (QSR International, Melbourne, Australia). Data analysis was conducted in a number of stages. After coding, two researchers (HO and GM) went over the emerging and sub themes to get a clearer impression of the strong themes emerging from the analysis. In the first stage, the theme on the use of hard liquor appeared strongly in the findings and study researchers were interested to further explore the roots of youth alcohol consumption. A closer examination of the codes showed alcohol initiation and its sub themes coming out in the study findings. Thereafter, two researchers (HO and GM) reviewed the emerging sub-themes regarding alcohol initiation in the coding scheme and further examined the data to assess it. In looking more closely at the findings, researchers then took time to reflect on both the physical and social aspects of alcohol initiation and noted the role of important social actors in youth’s first encounters with alcohol. The review of literature on youth alcohol use and explanations regarding the relationships between physical space, social relationships and social actors helped to shape and strengthen the analytical framework. Subsequently, a write up plan was drafted before the themes were written up.

### Ethical considerations

In order to obtain informed assent and consent before participation in the study, students were informed about the study and then invited to give written assent (if they were between 15 and 18 years old); or written consent (if they were aged 18 years or above). For day schools, an information sheet was provided to students to present to their parents a week before data collection. This sheet gave details to parents about the study and invited them to raise any questions or concerns they had, and to contact the investigators. This gave parents an opportunity to opt out of the study if they wished to do so. For boarding schools, this strategy was not possible due to communication limitations in Tanzania. In this case, we obtained permission from the responsible class teachers, including personal assent or consent. The mentioned strategies are stated in the study protocol and were cleared by the responsible ethics boards. Ethical clearance to conduct this study was acquired from The National Research Ethics Committee in Tanzania (NIMR/HQ/R.8a/vol. IX/1339) and the Ethics Committee of the London School of Hygiene and Tropical Medicine (LSHTM ethics ref: 6149).

## Findings

We conducted 35 IDIs among young people aged 15 to 24 years in Kilimanjaro and Mwanza regions. Seventeen IDIs were conducted in Kilimanjaro region (11 among males and 6 among females) and 18 in Mwanza (11 among males and 7 among females). Among the 35 interviewees, 12 were formal sector employees, 12 casual laborers, 5 secondary school students and 6 college students. Participant demographic and behavioral characteristics are shown on Table 1.

**Table 1:** Socio demographic and behavioral characteristics of study participants.

		Secondary school students		College and University students		Formal employees in industry		Casual laborers	
Characteristic	Response	Male	Female	Male	Female	Male	Female	Male	Female
Total sample	N	4	2	3	2	8	4	7	5
Region	Kilimanjaro	2	1	1	1	4	2	4	2
	Mwanza	2	1	2	1	4	2	3	3
Religion	Christian	4	1	2	1	4	2	6*	4
	Muslim	0	1	1	1	4	2	0	1
Education	Primary	n/a	n/a	n/a	n/a	2	3	4	5
	Secondary and above	4	2	3	2	5	1	3	0
	No formal education	n/a	n/a	n/a	n/a	1	0	0	0
Marital status	Single	4	2	3	2	7	3	6	2
	Married	0	0	0	0	0	1	1	2
	Divorced	0	0	0	0	1	0	0	1
Drinking status	Current drinker	1	0	1	1	3	1	3	0
	Former drinker	1	0	2	0	2	1	2	4
	Never drank	2	2	0	1	3	2	2	1

* = one participant did not respond.

For the majority of young people, first encounters with alcohol occurred during adolescence. Participants reported first consuming alcohol between the ages of 13 and 20 and a few reported first tasting or sipping alcohol as early as 10 years old. In reporting current drinking status, overall, more participants reported not drinking. However it is important to point out that, compared to those who do not drink, more participants reported ever consuming alcohol. In Kilimanjaro, more young people reported being current or former drinkers compared to those in Mwanza with the lowest reported use being among young women in Mwanza. Young men reported initiating alcohol earlier than young women and more young men reported being current or former drinkers. Themes surrounding young people’s initiation into alcohol consumption focused on two main parts; the social environment where alcohol consumption first took place and secondly, the interpersonal influences of first encounters with alcohol.

### The social environment (mesosystem influences)

Study participants discussed three main social environments or mesosystems where initiation of alcohol use largely took place; the home, at social or special occasions, and in stressful situations or environments.

#### In the home

The majority of young people described the home as being the place where they were first introduced to alcohol. The availability of alcoholic beverages in the home, in places known and accessible to the children, was considered an important trigger in the initiation of alcohol use among young people. One participant from Mwanza explained:

*Sometimes you find that parents have alcohol in the house*, *lots of alcohol*. *For example you may find that the refrigerator is full of alcohol*. *A lot of times*, *you find that a child can take [alcohol] and start trying [to drink]*. *They start by trying and then slowly get used to it*. *(*Female, Secondary School student, Mwanza)

Home breweries, where local brew is made and sold as a source of income for the household, also influence young people to begin tasting and consuming alcohol in the home. In such environments, alcohol is readily available and can be accessed discreetly by young people for example, through tasting in secret. Secretly tasting alcohol was described to open doors to alcohol use at a young age. A female participant reflected:

*In the village*, *you find that the mother or father brews alcohol [at home]…and the child is right there*. *When the brewing is done and they leave the room*, *the child would steal some and drink a bit*. *In end*, *they get used to the taste and become a drinker*. (Female, Informal sector, Mwanza)

#### Social events and special occasions

In both sites, alcohol was reported to be an important part of social celebrations. Young people reported that social events such as weddings, graduation parties, religious celebrations and holidays are among the most common environments where youth consume alcohol for the first time. While a few participants in Mwanza reported this, more than half of participants in Kilimanjaro recalled having their first drink at a local or family celebration. Social events were discussed to have a wide availability of alcohol and in some occasions, alcohol being more widely available than soft drinks. A number of participants reported first tasting and drinking alcohol in this context because of its wide availability. One male participant from Kilimanjaro recalled an Easter celebration:

*I*: *Perhaps, what made you drink that Easter holiday?*

*R*: *You find that*, *the environment on that celebration day was dominated by alcohol*. *So I was also tempted to use alcohol*. *Now when you use [alcohol] it is easy to also be tempted to drink again*. *Then you find that you’ve already become an alcohol drinker*. *(*Male, Secondary School student, Kilimanjaro)

#### The stressful environment

The idea that alcohol relieves stress was commonly discussed among study participants. Some study participants reported first consuming alcohol in what they considered stressful environments with hope that alcohol would help them overcome and cope with the situation. A male participant from Kilimanjaro described his experience:

*I had to perform in front of people you see* …*and there were many people that day*, *a lot of people*. *So because of that*, *it was hard for me to go [perform] in my right mind*. *So*, *I decided to drink alcohol for the first time*. *I had it in my mind that alcohol will help me overcome my nerves and it did…* (Male, Informal sector, Kilimanjaro)

Stressful environments were described to occur in numerous social situations. Participants described starting to drink alcohol because of stress due to family problems, being unemployed or suffering from other challenging economic circumstances. Some youth also described opting for stronger alcohol to have a quicker effect in order to deal with stress. One participant, who did not specify the cause of her stress explained:

*I: Why did you decide to first taste Castle [lager] while there was also Safari and Balimi beer*…*why Castle?*

*R*: *I drank Castle because I heard that it gets you very drunk and I was stressed*. (Female, Informal sector, Mwanza)

### Microsystem influences to initiation of alcohol use

#### Parents and relatives

Alcohol initiation, which was reported to occur in the home, was often described to be a result of influence from parents and relatives (siblings and extended family) who also consumed alcohol. With reference to personal experience and the experiences of other young people, participants discussed that children often model the drinking behavior of their parents particularly if the parents are heavy drinkers and if they drink in the presence of their children. Other participants also reflected on the role of parents in encouraging children to taste or drink alcohol when it is available in the home. This encouragement along with parents purposely feeding alcohol to young children was seen as an important factor in influencing children to start consuming alcohol at a young age. One participant from Kilimanjaro explained:

*I’ve seen a parent once give some alcohol to a three year old child*. *So*, *the child gets used to that alcohol*. *By the time they are six years old*, *they can finish an entire bottle*. *So*, *parents do play part… (*Female, Formal sector, Kilimanjaro)

Parents and relatives were also said to often send young children to purchase alcohol for them from numerous outlets, both local brew and industrial alcoholic beverages. The majority of participants held the view that going to purchase alcohol encouraged young people to begin secretly tasting alcohol and eventually getting used to it. One participant from Mwanza recalled his experience as follows:

*I had an uncle who used to send me to buy alcohol for him*. *Sometimes he would say*, *go buy gongo [local spirit] from mama so-and-so’s house*. *As usual*, *that’s where I learnt how to drink*. *If he left the room briefly*, *I would also drink some of it*. (Male, Formal sector, Mwanza)

#### Peers

Friends and peer groups were commonly mentioned as key influencers in first time alcohol use among young people in the study settings. Young people with friends that are alcohol users were likely pressured or influenced to also begin consuming alcohol in order to ‘fit in’ as part of the group. Compared to young women, more young men described peer pressure as the main influence for their initiation of alcohol use. Study participants explained that youth influence peers to start drinking through peer pressure or by peers modeling the drinking behavior of those who consume alcohol. This occurred during social events where alcohol was freely available with little restriction of drinking. One participant who began drinking because of peer influence recalled his experience:

*One of my friends invited me to a wedding*. *So we went and they first brought sodas to our table*, *then he told them to bring us beers instead*. *They brought us beers and he persuaded me by saying ‘drink at least one beer*, *it’s not bad’ so*, *I drank it*. *Then I drank another one*. (Male, Informal sector, Mwanza)

Another participant also explained the main reason why young people begin using alcohol as follows:

*It is mostly because of curiosity…and copying the behavior of those who already drink alcohol…* (Male, Secondary school, Mwanza)

#### Intimate partners

Young people’s intimate partners, both male and female, were reported to play part in the initiation of alcohol use. Spouses were mentioned to play part in convincing and at times pressuring their significant others to consume alcohol during outings. Influence from intimate partners to consume alcohol was connected to having unprotected sex and multiple sexual partners. A female participant who became pregnant because of having unprotected sex after frequent alcohol use with her partner spoke about how she started using alcohol:

*When I met my son’s father*, *he convinced me to go out drinking with him so we used to go to discos [clubs]… it came to a point where I told him I could not cope with that life and he told me if I did not want to go out to clubs and drinking then we had to break up and I said fine*. *When he started calling me again…that’s when I learned how to drink alcohol… if he bought me a soda*, *he would take his Konyagi [liquor] bottle and pour some of it in my drink…at that time*, *I had just finished class seven [primary school]…* (Female, Informal sector, Kilimanjaro)

Young women who consumed alcohol were equally mentioned to influence their male partners to start drinking alcohol if they discovered that they did not previously consume alcohol. A male participant shared that:

*Yes*, *I have a friend who drinks…*.*he started drinking because of women*. *He met a woman*, *at that time he was not a drinker* …*that woman used to ask him why he did not drink alcohol while she drank and he said he would start*. *The woman persuaded him to start drinking and he did…*.*then he also started having a lot of women [at once]…* (Male, Secondary School, Kilimanjaro)

## Discussion

Our study findings illustrate young people’s drinking behaviors and their recollection of initial encounters with alcohol use. Our findings show that young people in these two regions mainly begin drinking at home, during social events or occasions, and in stressful environments or situations. In these settings, the main influence for initial alcohol use comes from parents and relatives, peers and intimate partners. We also found that, young people start taking alcohol during adolescence often in their early teens. However, tasting or sipping alcohol begins as early as at ten years of age. Compared to young men, young women were less likely to drink and initiated drinking later. Other studies have also reported similar findings [13, 14, 25]. In these findings, we see the social space assuming a more dominant role compared to physical space as the primary influence in the initiation of alcohol use among young people.

In line with Bourdieu’s definition of social space, our findings illustrate the primacy of existing social relationships with other actors in influencing young people’s first drinking experiences. Although some studies have shown that the physical space is central to youth alcohol initiation and use [26], our findings show that it is the existing sets of social relationships in these physical spaces that have the primary influence on the initiation of alcohol use. The way young people relate to important social actors in their daily lives; mainly their parents, peers and intimate partners, ultimately determines how, when and under what circumstances they choose to start drinking alcohol. These social actors are persuasive and play part in influencing youth’s behavior including that which relates to their use of alcohol. In this regard, social space is the embodiment of these sets of relationships that consequently influence alcohol initiation among the population.

The wide availability of free alcohol in the home and at social events provides easy access and the physical environment for early alcohol consumption among youth. However, the ways in which youth interact and relate with others within these locations is what facilitates their initiation of alcohol use. From our findings, young people’s first interaction with alcohol is mainly from family members and peers. Social persuasion is evident in efforts from peers and parents to encourage youth to try their first alcoholic drink. Parents who encouraged youth to ‘take a sip’ and peers who urged friends to ‘try one beer’ played part in encouraging drinking and demonstrated their own use of alcohol in the process [27, 28]. In this regard, social persuasion—with underpinning power relations (such as in parent child relationships)—is important in influencing young people’s decisions and actions to start using alcohol. Whether in the home or at social gatherings such as weddings and religious ceremonies, it is apparent that restrictions on the minimum legal age of drinking are less applicable in these social spaces [29] compared to how it is considered and implemented in drinking establishments such as bars. All these factors contribute to creating social spaces where young people can start tasting and consuming alcohol with minimal normative or legal restrictions.

The home is an important space in interventions addressing youth alcohol use and initiation as some studies have found it to be the primary location for alcohol consumption [29]. As observed from our findings and other similar studies, alcohol is often perceived differently in the home compared to how it is perceived in the public legal environment [29]. Particularly, in homes where alcohol is sold as a commodity, it is treated and considered as an ordinary product for sale that contributes to the household income [30]. As a result, youth in these spaces become acquainted to alcohol as a ‘normal’ commodity.

One study also noted that parental provision of alcohol in the home is often not accompanied by precautions on how much drinking is considered harmful [31]. Participants in our study also did not mention precautions from parents regarding harmful drinking at initiation, however we did not specifically ask about this. Precaution from parents regarding harmful drinking is important to note as, some studies on adolescent drinking have found that parental provision and supervision of alcohol use among adolescents may not protect them against risky drinking and other alcohol-related problems [31, 32].

Peer and parental influence were the most commonly reported drivers of first time alcohol use among young people, peer influence being the strongest particularly among young men. Depending on the nature of their social space, young people can be easily influenced to use alcohol when it is part of their daily interaction with their peers. Based on our findings, stronger peer pressure to consume alcohol among young males could partly explain the higher reported rates of initiation and current consumption among young men compared to young women on both study sites. Other studies on alcohol use among youth show that drinking among peers influence alcohol use among their non-drinking peers whereas, a common disapproval of drinking among peers is linked to less alcohol consumption [33].

The use of alcohol at social events and special occasions in African settings can be traced back to traditional ceremonies which utilized alcohol as part and symbol of celebration and entertainment [34]. However, in the past, drinking was regulated through social control over when people (including youth) drink, how to drink and with whom [34]. Currently young people are still socialized to view alcohol as part and parcel of social celebrations and entertainment, but with much less social control. From our findings, it seems this is more so the case in Kilimanjaro than in Mwanza, which implies an acceptability of underage drinking and account for the higher number of participants in Kilimanjaro who reported initiating alcohol and being current drinkers. In the past, during traditional communities, alcohol was consumed as a symbol of interaction and cohesion [34], youth (especially males) in this study referred to the desire to use alcohol with the purpose of intoxication [35]. In order for interventions addressing youth alcohol use to be effective, there is a need to understand young people’s individual knowledge about alcohol, motives for using alcohol and meanings they attribute to it.

The use of alcohol as a stress reliever has also been discussed in the literature [36]. However, in this context, there is a need to learn more about the sources of the young people’s individual knowledge and attitudes about alcohol as a stress reliever. There is also a need to explore more concerning how and in what situations stress influences young people to start drinking and the role of social interaction in facilitating this form of drinking. In addressing this association between alcohol use and stress relief, interventions could encourage young people to derive other mechanisms of coping with stress that are less harmful [37].

Our findings show that, young men and women play part in influencing their intimate partners to start consuming alcohol. We also found that, in some intimate relationships, alcohol plays an important part in initiating or maintaining a romantic relationship. This highlights the role of pressure from partners to start alcohol use, whether they are a peer or older partner [38]. As our findings suggest, alcohol in this social space contributes to young people’s engaging in risky behaviors such as multiple partnerships and unprotected sex. Social spaces where alcohol use takes place, such as bars and clubs are the same settings where sexual relationships are initiated [39]. In such spaces, alcohol is used as a means to facilitate interaction and create an informal mood. Interventions aiming at delaying initiation of alcohol use among young people need to address societal drinking norms in their totality and how they affect young people’s health.

Our study has some limitations. Participants’ retrospective accounts of their first use of alcohol may be subject to recall bias particularly among those who began consuming alcohol at a very young age. However, we are confident that accounts provided by participants were likely accurate as personal experiences were cited and described in detail. Participants who failed to recall this information were open about it by stating their failure to recall the specific situation or time when they had their first drink. Secondly, social desirability bias is also a concern as alcohol consumption is a sensitive issue. Therefore, there could be underreporting of alcohol use, especially among young women. On the other hand, there is possibility of over reporting among young men as it compliments masculine behavior [40]. To address some of these limitations, we ensured that same sex interviewers conducted the interviews.

## Conclusions

Our study shows that young people often begin consuming alcohol during adolescence. Social interactions and environments encourage alcohol consumption in combination with influences from interaction with significant ‘actors’ in this space such as parents, peers and intimate partners. Our findings clearly demonstrate that interventions addressing alcohol use among youth need to address the social space in which consumption occurs and engage the socially significant actors in this space. Promotion of messages and strategies targeting the various social spaces could be as effective in addressing alcohol use among youth. There is a need to further explore underlying societal drinking norms to better understand how these norms shape influential social environments and their role in young people’s interactions with their family and peers.
